# The Urgency of Conducting Serological Studies for COVID-19

**DOI:** 10.34172/jrhs.2020.14

**Published:** 2020-05-30

**Authors:** Ghobad Moradi, Ehsan Mostafavi, Ali-Akbar Haghdoost

**Affiliations:** ^1^Social Determinants of Health Research Center, Research Institute for Health Development, Kurdistan University of Medical Sciences, Sanandaj, Iran; ^2^Department of Epidemiology and Biostatistics, Research Centre for Emerging and Reemerging infectious diseases, Pasteur Institute of Iran, Tehran, Iran; ^3^Modeling in Health Research Center, Institute for Futures Studies in Health, Kerman University of Medical Sciences, Kerman, Iran

**Keywords:** COVID-19, Seroepidemiologic Studies, Epidemiology, Enzyme-linked immunosorbent assay

## Abstract

**Background:** COVID-19 has been the most priority of the world since the early 2020s. We aimed to investigate the importance, urgency and value of serological tests for monitoring and evaluation of COVID-19.

**Study design:** Rapid review.

**Methods:** This study was conducted through a review of seroepidemiological studies to evaluate their strength and weakness in monitoring and predicting the epidemic situation of COVID-19.

**Results:** Conducting serological studies is an important measure to determine the status of the COVID-19 in affected countries. These studies may also be used to estimate cumulative incidence of the disease, and to get an impression about the level of the epidemic.

**Conclusion:** If an accurate serological test is available it can be used for seroepidemiological studies and epidemic investigation in special context, but given the current situation, it may not be possible to be used for screening the normal population and in care and treatment. This research highlighted the importance and urgency of conducting serological studies for monitoring the COVID-19 situation and evaluation of the interventions.

## Introduction

A little over four months after the first report of COVID-19, the disease became the world’s first and utmost health priority. It has had a deep and vast impact globally. It is not a hazard for human health only, but its effects on the economy in micro and macro-levels, security, social solidarity, politics and international relationships are also considerable ^1^. Failure to control the disease can ensue epidemic peaks that hospitals may not be able to handle. Under such circumstances, creative and timely measures are required to design effective interventions^2^. The actual number of patients in communities is much higher than the officially reported number of cases^3^.

We aimed to investigate the importance, urgency and value of serological tests for monitoring and evaluation of COVID-19.

## Methods

This study was conducted through a rapid review of published references on the seroepidemiological studies for monitoring and predicting the epidemic situation of COVID-19.

## Results

**Figure 1:**
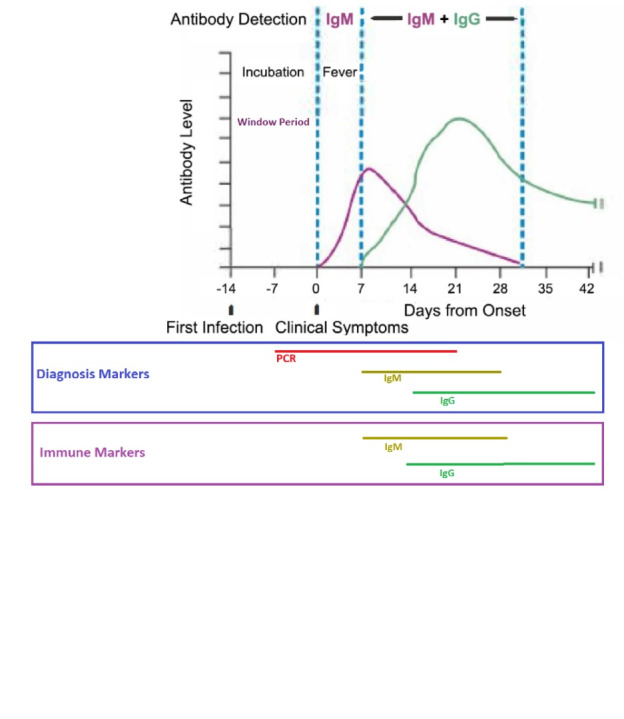
The natural history of COVID-19 in terms of the appearance of symptoms, the diagnostic and immunological markers ^5^

Antibodies may not be detected in the early days of infection. This limits the effectiveness of COVID-19 serological tests, hence the fact that these tests should not be considered as the sole basis for diagnosing COVID-19. The level of IgM antibody begins to rise one week after the initial infection while IgG appears later (usually within 14 d following the first signs of infection) (Figure 1)^4,5^. The asymptomatic or mildly symptomatic patients may show low-titer antibodies that may affect the sensitivity of the serological tests^6^. About 30% of people show very low antibody titers, and around 5% may have undetectable antibody titers ^7^.

The time of antibody production and the strength of antibody response depends on several factors, including age, nutritional status, the severity of the disease, and certain medications or infections that suppress the immune system such as the underlying diseases. For this reason, some affected people may show negative test results (false negative). Detection of those highly exposed to the virus without any symptoms and raise of antibody can help to explore the level of susceptibility of subjects and its determinants. Serological tests may also cross-react with other pathogens, including other human coronaviruses, and give false-positive results ^8^. To adjust the observed percentage of positive results for the sensitivity and specificity of results, a few formulae can be used to estimate the positive percentage of the cases in a community ^9^.

**Figure 2:**
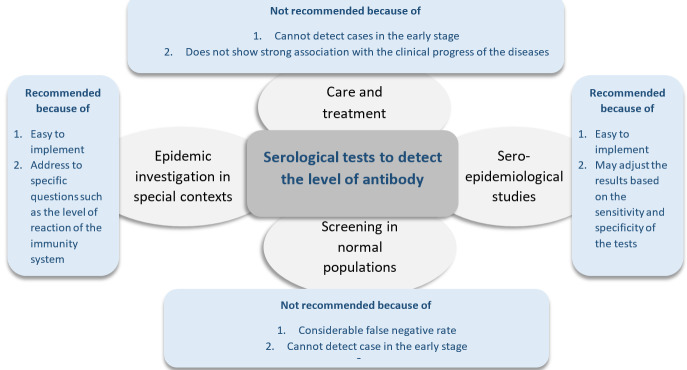
Applications of serological tests to study various aspects of COVID-19

If an accurate serological test is available, it can be used for epidemic investigation in a special context such as nursing homes, prisons, etc. ^10^,and for seroepidemiological studies. Late raise of antibodies and even undetectable level of antibodies are the main barriers to recommend these tests as an alternative method for screening the normal population and in care and treatment (Figure 2).

In seroepidemiological studies, specifying the ratio of those with positive IgM or IgG alone or both can help determine the epidemic status of COVID-19 in recent days and weeks. Normally, as the duration of IgM positivity is shorter than IgG, and duration of both of them to be positive is shorter, so if the incidence rate is uniform, the lowest positive percentage should be reached for the concurrence of both IgM and then IgG, then positive for IgM and then positive for IgG. A high ratio of people with positive IgM can indicate that the virus and disease are active in the community. However, a high proportion of people with positive IgG can be indicative of an epidemic in the later stages of the disease. On the other hand, the high concurrence of positive IgG and IgM can contribute to interpreting the status of the epidemic in a community.

## Discussion

A quick and extensive serological study on targeted groups can be conducive to gaining access to valuable information in affected communities. The results of such studies can facilitate the implementation of interventions tailored to each group, and to determine the most optimal time to reopen businesses, schools and universities. Conducting these studies at different frequencies helps obtain a better picture of the disease.

These tests can also be useful for public health professionals and clinicians to estimate the cumulative incidence to personalize the risk of developing the disease over time. Cumulative incidence is calculated as the number of new cases of COVID-19 detected by serological tests divided by the total number of individuals in the population at risk of infection since the start of the epidemic.

In case of limited access to antibody kits, a high priority should be given to studying high-risk groups susceptible to infection, including health care workers and family members of infected people.

In seroepidemiological studies, where IgM is detected in the studied population, the results can also be used as a screening method because the disease may still be in the active phase in these positive cases. Identifying IgM positive cases and performing PCR to confirm their current disease can be a strategy to find some new cases. As the world is currently focusing on vaccine production as an effective approach to controlling the disease, it is also possible to evaluate the effectiveness of vaccines by measuring the level of antibodies in vaccinated people.

When at least 70% of the population is immune to COVID-19, this provides indirect protection, or herd immunity, to those who are not immune to the disease. Key to understanding this issue is the seroepidemiological study of COVID-19 in different population groups.

Seroepidemiological studies can be further utilized to make decisions about bringing employees back to work. People who have acquired enough immunity can receive a health certificate to return to work with a higher level of assurance^10^. A significant proportion of people may be infected with the asymptomatic form of the disease; therefore, acquiring information concerning the immunity of different groups of people at high risks of occupational exposure, such as health care workers, can help the infected individuals to continue their work more confidently.

## Conclusion

It is necessary to urgently obtain up-to-date information about the disease to be able to tackle it. The cumulative incidence of the disease is one of the most important factors for specifying its status. If this indicator is set correctly in a community, more targeted and appropriate interventions can be designed and the results can be evaluated more accurately. Lack of access to valid kits is one of the limitations hindering the studies of such ilk.

Because of the level of accuracy of the serological tests, still, it is not recommended to be used in the care and treatment of subjects and the screening of patients. There are a few days lag between contracting the infection and the appearance of antibody in the blood, which significantly reduce the validity of these tests in detecting subjects in the earliest phase as a tool for screening. Besides, the considerable proportion of false positives and false negatives, and the undefined association between the excretion of the virus and the level of antibodies are concerning points which limit the application of these tests in the process of care and treatment of the patients. However, without any doubt, they are a powerful tool to assess the intensity of the transmission of the infection in a community in epidemic investigations.

All affected countries should promptly provide the necessary support for the production of serological kits with high sensitivity and specificity. Immunologists and molecular biologists can help human communities via conducting research on the natural history of the disease, determining the time of antibody production, and identifying its diagnostic and immunological markers.

## Conflict of interest

The authors declare that there is no conflict of interest.

## Highlights

- There is a great and prompt need for serological tests with high sensitivity and specificity for COVID-19.
- Conducting serological studies at different periods help to obtain a better picture of the actual situation of COVID-19.
- The serological tests are not still recommended to be used for screening the normal population and in care and treatment.
